# Utilisation of Intensive Foraging Zones by Female Australian Fur Seals

**DOI:** 10.1371/journal.pone.0117997

**Published:** 2015-02-18

**Authors:** Andrew J. Hoskins, Daniel P. Costa, John P. Y. Arnould

**Affiliations:** 1 School of Life and Environmental Sciences, Deakin University, 221 Burwood Highway, Burwood, Victoria, 3125, Australia; 2 Center for Ocean Health, University of California Santa Cruz, 100 Shaffer Road, Santa Cruz, California, 95060, United States of America; University of Western Australia, AUSTRALIA

## Abstract

Within a heterogeneous environment, animals must efficiently locate and utilise foraging patches. One way animals can achieve this is by increasing residency times in areas where foraging success is highest (area-restricted search). For air-breathing diving predators, increased patch residency times can be achieved by altering both surface movements and diving patterns. The current study aimed to spatially identify the areas where female Australian fur seals allocated the most foraging effort, while simultaneously determining the behavioural changes that occur when they increase their foraging intensity. To achieve this, foraging behaviour was successfully recorded with a FastLoc GPS logger and dive behaviour recorder from 29 individual females provisioning pups. Females travelled an average of 118 ± 50 km from their colony during foraging trips that lasted 7.3 ± 3.4 days. Comparison of two methods for calculating foraging intensity (first-passage time and first-passage time modified to include diving behaviour) determined that, due to extended surface intervals where individuals did not travel, inclusion of diving behaviour into foraging analyses was important for this species. Foraging intensity ‘hot spots’ were found to exist in a mosaic of patches within the Bass Basin, primarily to the south-west of the colony. However, the composition of benthic habitat being targeted remains unclear. When increasing their foraging intensity, individuals tended to perform dives around 148 s or greater, with descent/ascent rates of approximately 1.9 m•s^-1^ or greater and reduced postdive durations. This suggests individuals were maximising their time within the benthic foraging zone. Furthermore, individuals increased tortuosity and decreased travel speeds while at the surface to maximise their time within a foraging location. These results suggest Australian fur seals will modify both surface movements and diving behaviour to maximise their time within a foraging patch.

## Introduction

The ability of animals to identify and efficiently utilise profitable foraging habitat is critical, not only for their survival but also for optimizing reproductive success [1]. In offshore marine environments there are high levels of temporal and spatial heterogeneity in the distribution of resources [2]. Predators within this environment will often focus foraging in areas where prey can be more reliably found such as frontal zones, upwellings, seamounts or prey spawning grounds [3–8]. As such, it is advantageous for marine predators to remain within or near these productive areas for as long as possible [9,10]. However, throughout periods of parental care, land-breeding marine predators such as seabirds and seals will often have to adopt a central placed foraging strategy, returning to colonies at regular intervals to provision young [11]. Consequently, these individuals have a particular need to efficiently locate and then utilize high quality foraging patches in order to maximize the delivery rate of food to their offspring

Studies investigating the foraging behaviour of seals and seabirds have identified relationships between their foraging behaviour and areas where prey availability is expected to be relatively high [6–8]. In these areas, individuals have been recorded to increase residency times by displaying searching movements in their surface behaviour (area-restricted search, recorded as reduced surface speed and increased tortuosities of tracks) and within their sub-surface behaviour (diving bouts, recorded as temporally discrete patches of increased diving activity) [12–15]. However, the majority of these studies have investigated this behaviour in pelagic foraging species, many of which forage in open water off the continental shelf where prey distribution is influenced by large-scale drivers of productivity [6–8,12–15]. In contrast, fewer studies have investigated how benthic foragers alter their behaviour while in productive feeding areas [16–20]

The environment encountered by benthic foraging species is very different to their pelagic counterparts, as such their behavioural responses to prey availability may also differ. While pelagic foraging species are able to search for prey throughout an entire dive as well as at the surface, benthic foraging species can only commence searching once their maximum depth has been reached [21]. This results in individuals having to spend a larger proportion of the dive during the bottom phase, culminating in overall longer dives that are more likely to exceed their aerobic dive limit [22]. The different foraging modes also result in animals encountering differing prey. Pelagic species target smaller prey which may occur in dense aggregations that are highly spatially and temporally heterogeneous [21]. In contrast, benthic foraging species target a less productive habitat and focus on larger prey that are distributed less heterogeneously [21,23]. Overall, this implies that benthic foraging species are buffered from the temporal variability in prey concentrations that pelagic species encounter, but will on average have to work harder than pelagic foraging individuals for similar energetic gains, despite the greater reliability with which their prey are encountered [21,22].

The Australian fur seal (*Arctocephalus pusillus doriferus*) is a species endemic to the shallow continental shelf waters of Bass Strait, Australia [24,25]. Previous studies have identified it as being a primarily benthic foraging species that forage in several broad foraging ‘hotspots’ within Bass Strait [24,26]. Within these areas, they forage on a variety of different species of fish and cephalopods, including redbait (*Emmelichthys nitidus*), jack mackerel (*Trachurus declivis*) and arrow squid (*Notodarus gouldi*) [27,28]. Pups are born in November/December and are nursed for approximately ten months before being weaned [29]. During this time, females adopt a central place foraging strategy travelling on average 122 ± 19 km from their colony and spending an average of 6.7 days at sea before returning to provision their pups [24,26]. While at sea, they dive repetitively for extended periods of time (up to 36 hrs) with no known bout structure [26]. Although coarse-scale information is available for female Australian fur seal at-sea locations [24,25] and diving behaviour [26,30], it is not currently known how females spatially distribute foraging effort or if they modify diving/foraging behaviour during different stages of their foraging trip.

The combination of tracking and diving data are capable of providing greater information about an individual’s ecology than either of the two methods alone [16,31,32]. While the majority of diving behaviour analyses give an indication of sub-surface movements, it is done over a two dimensional time-series and does not indicate how the animal behaved spatially [33–36]. Furthermore, although surface movements may give an indication of an animal’s horizontal movements, it does not completely represent their behaviour under the water [37]. Indeed when looking at the foraging behaviour of southern elephant seals (*Mirounga leonina*), Bailleul et al [31] discovered that using diving behaviour and tracking data together in a first passage time analysis resulted in an overall reduction in the size of the search areas identified, suggesting an increased resolution when compared to analyses using movement data only. Currently, it is unknown if solely tracking data can be used to identify areas of increased foraging intensity for Australian fur seals.

In this study, we aimed to investigate within a benthic foraging species (the Australian fur seal): 1) the effect of introducing diving behaviour into the detection of areas of increased foraging effort, 2) the spatio-temporal distribution of foraging effort; and 3) the behavioural modifications that characterise increases in foraging effort.

## Methods

### Ethics statement

All work was carried out with approval of the Deakin University Animal Ethics Committee and under Department of Sustainability and Environment (Victoria, Australia) Wildlife Research Permit (10005848). Kanowna Island is part of the Wilsons Promontory Marine National Park and was accessed under permit from Parks Victoria.

### Animal handling and instrumentation

The study was conducted on Kanowna Island (39° 09’ S, 146° 18’ E), central northern Bass Strait, south-eastern Australia, during the austral winters of 2006–09. The 30 ha island is host to a large breeding colony of Australian fur seals with an annual pup production of approximately 3400 [38]. A total of 44 adult females nursing pups were selected at random and captured using a modified hoop net (Fuhrman Diversified, Seabrook, Texas, U.S.A.). Once restrained they were given an intramuscular injection of the sedative Midazolam (approx 0.1 mg·kg^-1^) prior to induction of isoflurane gas anaesthesia delivered via a portable vaporiser (Stinger, Advanced Anaesthesia Specialists, Gladesville, NSW, Australia.; [39]). Anaesthetised individuals were removed from the capture net and weighed on a platform with a suspension scale (± 0.5 kg) and morphometric measurements (straight-line length, axillary girth, fore-flipper length) were taken using a metal tape measure (± 0.5 cm). A FastLoc GPS data logger (F1G; Sirtrack Ltd, Havelock North, New Zealand), time-depth recorder (MK10; Wildlife Computers, Redmond, WA, U.S.A.) and VHF transmitter (Sirtrack) were then glued in series to the pelage along the dorsal mid-line, just posterior to the scapula, using quick-setting epoxy (Accumix 268, Huntsman Advanced Materials Pty Ltd, Deer Park, Vic, Australia) and individually numbered plastic tags (Super Tags, Dalton Supplies, Woolgoolga, NSW, 2456, Australia) were inserted into the trailing edge of each fore-flipper. The FastLoc data loggers were programmed to sample at a minimum interval of 15 min while the animal was at the surface and the TDR was set to sample at intervals of 1 second for all times the animal was at sea.

Following completion of instrumentation procedures (usually within 45 min of capture), individuals were allowed to recover from the anaesthetic and resume normal behaviour. They were then recaptured as previously described after one or more foraging trips to sea and the data loggers were removed by cutting the fur beneath them with a scalpel before being re-released.

### Data processing and statistical analyses

Upon recovery of the devices, the data were downloaded to a portable computer and processed. The data from the GPS loggers were accurate however some highly erroneous locations still existed and to remove these, data were filtered using a basic speed filter [40]. After filtering, basic foraging metrics were calculated for each individual (trip duration, total distance travelled, mean speed, mean bearing) and GPS locations were linearly interpolated along each foraging track to be spaced evenly at 10 min intervals for use in later analysis.

TDR data were analysed using the diveMove package [41] in the R statistical environment [42]. Dives and their phases (descent, bottom, ascent and post-dive phases) were identified and characterised in terms of duration and maximum depth acheived. In addition to this, dives were classified into either benthic or pelagic using a custom written routine whereby individual dives were scored based on the proportion of time spent at the bottom of the dive multiplied by the maximum depth achieved during the dive. A kernel density estimate of the resulting score reveals a bimodal distribution, values to the left of the nadir between the two modes were taken to represent pelagic dives and values to the right of the nadir, benthic dives.

The spatio-temporal distribution of foraging effort was investigated using two methods. Firstly, areas of nominally high foraging effort, identified from spatial movement data only, were determined using a traditional first-passage time (FPT) analysis [43]. FPT refers to the time, *t*, taken for an animal to cross, from a location, a circle of radius *r* centred on that location. Initially, an animal’s spatial scale is calculated by determining the variance in FPT as a function of increasing radii. The radius that corresponds to the peak variance in FPT is then used as the spatial scale in subsequent analysis to calculate *t(r)* along the foraging time-series. If an animal is performing localized searching behaviour along the track this should be detected as areas of increased *t(r)* (refer to [43] for more detail). Female Australian fur seals have been known to haul-out at areas away from their breeding colony [25] so the FPT analysis was modified to exclude periods of time spent on land during a foraging trip.

Secondly, to incorporate diving behaviour, a modified first-passage time analysis (hereafter referred to as First Passage Diving, FPD) was developed. This analysis substituted *t(r)* with the amount of time an individual spent underwater in an area, *d(r)*, (i.e excluding time spent at the surface from the analysis). As with normal FPT analysis the spatial scale was first determined as the radii corresponding to the peak variance of time underwater. This was then used to represent *d(r)* for the different locations along the foraging track.

The locations for which FPT and FPD were determined were the linearly interpolated points spaced at ten minute intervals along the foraging tracks. The calculated spatial scale was determined separately for each individual, to allow for individual variations in searching behaviour for both FPT and FPD analyses. Then to accommodate comparisons between the methods FPT and FPD scores were scaled (between 0 and 1) for each individual. This resulted in two time-series of foraging intensity (measured by FPT and FPD) along the foraging track. To measure the degree of geographic overlap between these two, Bhattacharyya’s affinity [44] was calculated for each individual using the two complete foraging intensity time-series and the segments representing the greatest (scores above 0.8) foraging intensities (Equation 1).

$$OL=\sum_{i=1}^{n}\sqrt{\left(FPT_{i}\times FPD_{i}\right)}$$ (1)

This index measures the degree of overlap, *OL*, between two sample populations by multiplying the values in population 1, *FPT*, with the corresponding points in population 2, *FPD*, and taking the squared root of the resultant value. These values are then summed to produce a value between 0 and 1 with a score of 0 meaning no overlap between the two distributions and 1 meaning complete overlap. Furthermore, differences between the two methods in the proportion of time spent underwater within the areas of highest calculated foraging intensity were assessed using a student’s t-test. Kernel density plots were constructed from the aggregated FPD scores to highlight geographical regions of increased foraging intensity. Individual FPD scores were overlaid onto an evenly spaced grid (1 km^2^ grid cells) and the mean FPD score within each cell was calculated. This grid was then used to create a weighted kernel (weighted on mean FPD within each grid cell) using the fields package (ver 6.6.3) in R. The number, size and locations (distance and bearing from the breeding colony to centroid) of core foraging locations (areas encompassing the top 10% of the kernel estimate) were calculated and compared to the core home-range areas calculated with just the GPS locations.

Analysis of foraging behaviour was undertaken to investigate how individuals modified their activity (diving and movement patterns) in relation to the FPD characterization of the foraging trips. The FPD calculated foraging intensity scores were assigned to each dive using linear interpolation and then a regression analysis approach was used whereby the response variable was the foraging intensity score. Seven variables of summarized diving behaviour, dive duration (s), duration of bottom phase of dive (s), the mean descent/ascent rate (m·s^-1^), post dive duration (s), the total vertical distance travelled during the bottom phase of the dive (m. an indicator of prey chase behaviour, [45]), the type of dive (benthic or pelagic) and the maximum depth achieved (m) were considered as predictor variables for the analyses. A further two predictor variables describing spatial movements, namely fractal dimension (an index of the tortuosity of the track, calculated using the fdim ver 1.0–7 package), and mean travel speed (m·s^-1^) were included. A population level spatial scale was calculated as the peak in FPT variance averaged across all individuals and used in these calculations to remove any bias that may occur from calculating these measures from different sized areas of tracks between individuals.

Collinearity between predictor variables was assessed through comparisons of correlation coefficients and variance inflation factors (VIF) using the AED package (ver 1.0). Pairs of variables with VIF scores above 3, or one of the two vairiables for pairs that were highly correlated (Pearson’s correlation; *r* ≥ 0.7 or ≤ -0.7), were removed sequentially until all remaining predictor variables had VIF scores below 3 [46].

Initial inspection of the response variables against the predictor variables revealed a non-linear relationship in the data. As such, Generalised Additive Mixed effects Models (GAMMs) were fitted to the data using a Gaussian distribution, with an identity link function (mgcv ver 1.7–22). Smooth terms were fitted using penalised thin plate regression splines to all predictor variables except dive type. Dive type was categorical and was thus treated as a categorical factor within the models. Individual animal was used as a random effect and heteroscedascity within the model was accounted for using an exponential variance function. Model selection was performed using a step-wise backwards selection process whereby the least significant term in the model (selected by AIC) was rejected and the remaining variables were re-run through the model until all remaining parameters were significant.

## Results

Due to equipment failure or loss, complete records of at-sea movements and dive behaviour were sampled from 29 of the 44 individuals initially equipped. Mean body mass and length of individuals were 75.9 ± 11.6 kg and 152.5 ± 8.1 cm, respectively. The number of foraging trips covered by the deployments ranged from 1–12 trips per individual (2.7 ± 3.0). To remove the potential for bias from individuals with records of multiple foraging trips, only the first foraging trip of each individual was used in further analyses.

**Fig 1:**
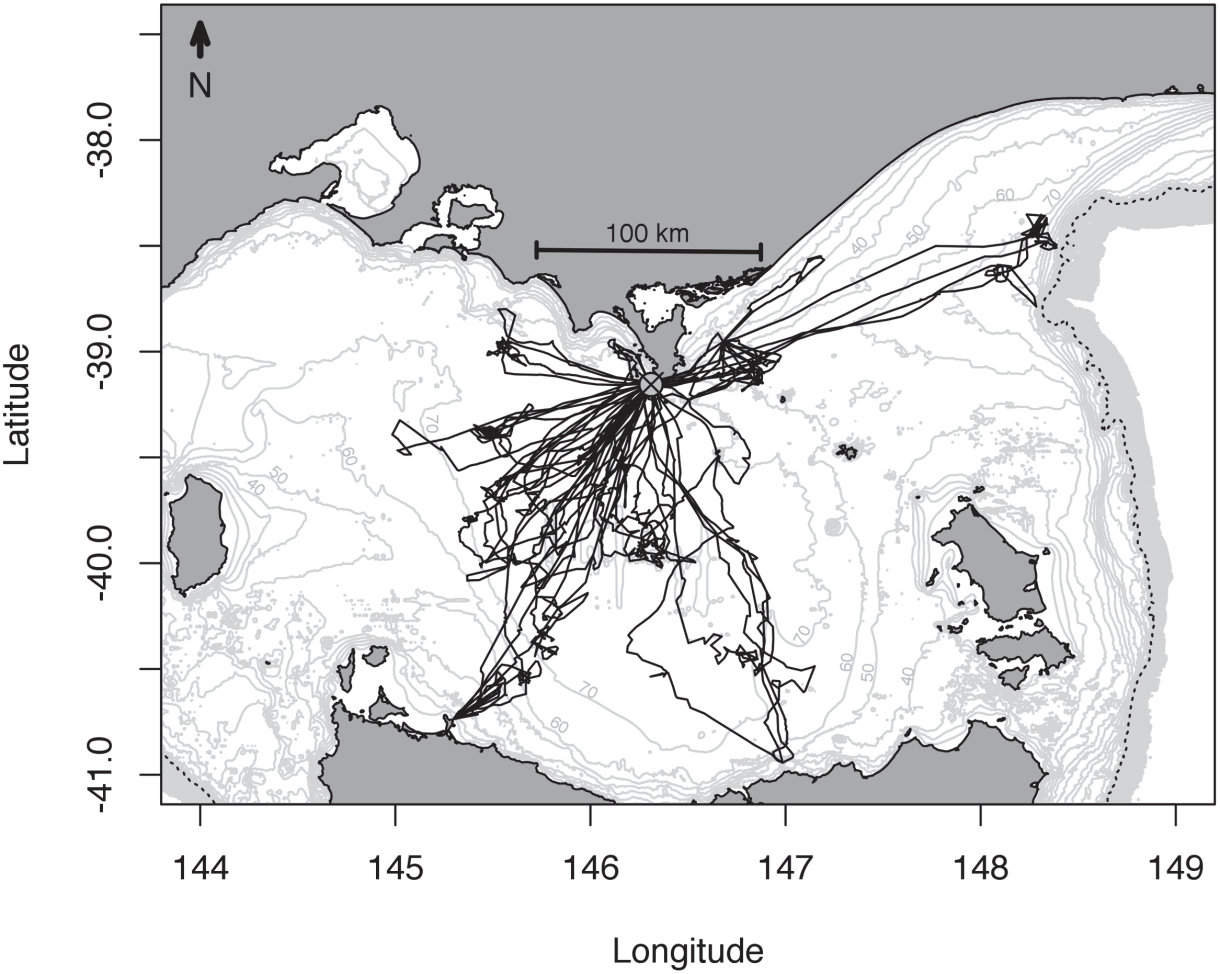
Foraging tracks of female Australian fur seals foraging from the Kanowna Island breeding colony, Bass Strait, Australia. Kanowna Island indicated by the grey circle with a cross through it.

Foraging occurred entirely within Bass Strait, with 84% of animals foraging in an area 92 km to the south-west of Kanowna Island, within the central Bass Strait basin (Fig. 1). On average, individuals travelled 51 ± 3 km·day^-1^, achieving maximum distance of 118 ± 50 km from the colony, during foraging trips that lasted 7.3 ± 3.4 days (Table 1; Fig. 1). Diving behaviour was consistent with previous studies [26], with animals exhibiting a primarily benthic mode of foraging (85 ± 13% benthic dives) (Table 1). Individual dives lasted a mean of 3.1 ± 1.1 mins, with the majority of dives reaching a depth 75 m (Table 1).

**Table 1:** Summary tracking/diving statistics and FPT/FPT overlap scores for female Australian fur seals from the Kanowna Island breeding colony, Bass Strait Australia.

ID	Duration (d)	Total Distance Covered (km)	Max Distance from Colony (km)	Speed (m/sec)	Bearing	Number of dives	Depth (m)	Duration (sec)	Maximum Duration (sec)	% benthic dives	Whole track	Core foraging areas
1	3.9	252.7	88.8	0.9 ± 0.8	177.6	702	85.2	195.1 ± 93.0	395	76	0.95	0.07
2	8.4	499.0	151.8	0.8 ± 0.6	157.9	1298	78.9	175.1 ± 45.5	305	96	0.98	0.27
3	4.8	311.3	96.7	0.9 ± 0.5	173.8	1055	83.1	134.7 ± 68.4	315	38	0.8	0
4	4.3	278.0	73.9	0.7 ± 0.6	76.7	1010	79.6	165.6 ± 83.2	490	78	0.87	0
5	3.7	320.9	121.7	0.9 ± 0.6	140.7	556	83.0	249.9 ± 66.8	430	95	0.92	0.39
6	6.6	310.4	93.5	0.8 ± 0.5	154.0	872	85.4	184.6 ± 70.5	421	85	0.64	0
7	4.2	109.4	53.3	0.5 ± 0.5	143.3	369	79.9	232.7 ± 66.2	385	91	0.67	0.04
8	4.2	249.4	117.3	0.8 ± 0.5	109.5	577	74.2	189.0 ± 80.5	350	68	0.85	0.15
9	2.6	192.7	78.0	0.7 ± 0.3	75.2	508	79.6	232.2 ± 78.1	390	88	0.95	0
10	6.9	313.5	118.6	0.7 ± 0.9	146.0	904	83.5	231.7 ± 74.3	581	98	0.71	0.06
11	8.9	539.6	207.1	0.9 ± 0.6	163.2	1280	80.2	191.4 ± 61.7	420	79	0.57	0
12	11.4	478.0	207.0	0.7 ± 0.4	161.9	1588	78.0	246.7 ± 64.2	490	94	0.68	0
13	5.2	54.4	16.3	0.2 ± 0.4	22.2	871	38.1	189.9 ± 37.8	295	97	0.96	0.69
14	5.8	276.1	121.0	0.7 ± 0.4	154.1	643	85.1	165.9 ± 43.6	310	94	0.67	0
15	4.5	311.3	131.1	0.8 ± 0.4	132.5	714	81.4	185.4 ± 47.5	300	97	0.8	0.05
16	11.7	622.8	196.0	0.4 ± 0.5	155.5	2201	48.0	142.5 ± 53.8	375	81	0.6	0
17	12.3	593.1	195.6	0.6 ± 0.5	67.4	2300	71.1	158.9 ± 73.0	365	82	0.96	0.52
18	14.8	616.7	83.5	0.6 ± 0.3	114.0	2704	82.0	157.0 ± 43.9	280	90	0.91	0.12
19	4.0	264.2	112.0	0.8 ± 0.5	132.7	708	79.7	215.2 ± 63.3	335	84	0.83	0
20	4.0	253.8	98.6	0.5 ± 0.5	166.2	582	83.4	168.0 ± 85.2	340	68	0.94	0.14
21	7.4	408.4	93.9	0.7 ± 0.4	172.3	1031	85.3	208.4 ± 55.1	370	88	0.81	0.01
22	5.3	222.9	89.3	0.4 ± 0.4	147.7	665	83.9	196.9 ± 55.0	330	95	0.64	0.01
23	5.6	245.9	93.7	0.4 ± 0.3	133.8	1043	79.0	184 ± 28.7	270	99	0.88	0
24	10.0	578.0	196.7	0.7 ± 0.5	69.4	2188	76.6	135.8 ± 68.8	385	65	0.87	0.02
25	13.3	646.0	59.5	0.6 ± 0.4	78.2	2443	61.4	158.1 ± 62.0	315	85	0.32	0
26	7.3	283.8	79.3	0.6 ± 0.4	68.5	1190	78.6	178.5 ± 73.9	315	79	0.6	0
27	8.6	255.4	101.1	0.4 ± 0.3	53.5	1932	26.3	144.5 ± 59.8	355	88	0.27	0
28	13.3	586.2	196.0	0.4 ± 0.3	155.7	1856	67.5	251.3 ± 82.4	610	94	0.72	0
29	7.6	366.7	159.8	0.6 ± 0.4	164.5	1518	81.7	173.3 ± 49.7	335	93	0.8	0
Mean	7.3	360.0	118.3	0.6	126.5	1217.5	75.2	187.7	374.3	85	0.77	0.09
SD	3.4	162.4	50.7	0.5	43.2	663.0	14.3	63.3	82.7	13.07	0.18	0.17

Data are presented as means ± standard deviation (where appropriate). Overlap scores, calculated by Bhattacharyya’s affinity and shown for whole tracks and core areas (FPT/FPD score > 0.9).

### Detection of intensive foraging zones

**Fig 2:**
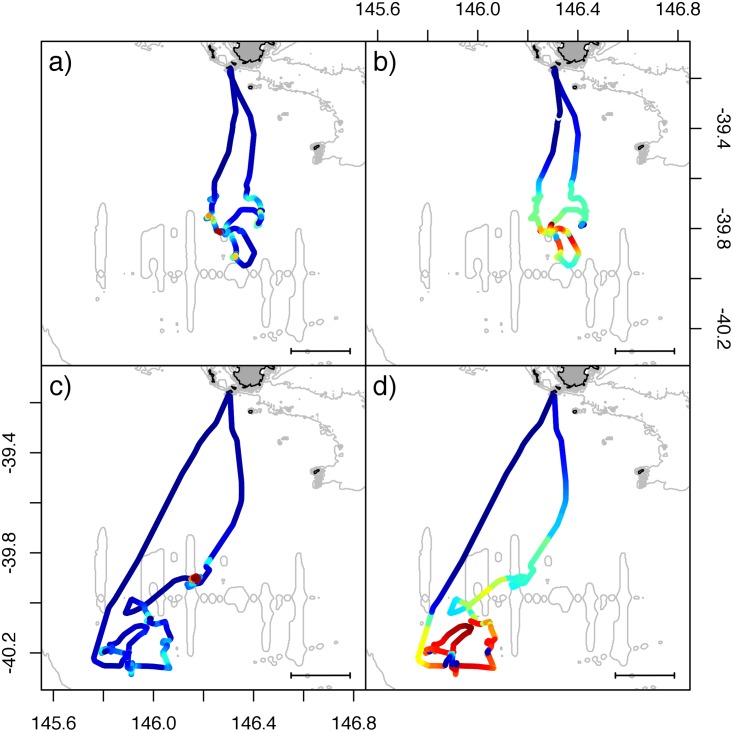
Comparison of geographic position of foraging intensity calculated using FPT (a and c) and FPD (b and d) from two individual female Australian fur seals foraging from the Kanowna Island breeding colony, Bass Strait, Australia. Color scale in panels a) and b) shows low (blue) to high (red) foraging intensity scores. Distance scales represent 20 km.

**Fig 3:**
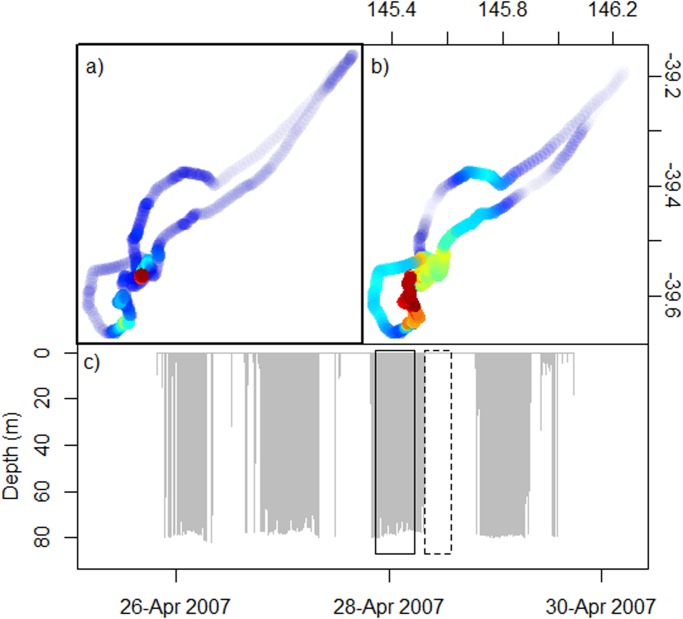
Comparison of a) FPT, b) FPD foraging intensity and c) the corresponding dive record for an individual female Australian fur seal foraging from Kanowna Island, Bass Strait, Australia. Color scale in panels a) and b) shows low (blue) to high (red) foraging intensity scores. Rectangles in panel c) show temporal position of core (red) foraging intensity scores for FPT (dashed) and FPD (solid) analyses shown in panels a) and b).

The FPT and FPD analyses detected areas of increased foraging effort (calculated as increased time or increased time-diving, respectively, in an area), however, the spatial scales calculated by FPT and FPD were not found to vary significantly between the two methods (Paired samples t-test: t_28_ = 0.696, p = 0.49). Comparison of the techniques revealed noticeable geographic differences in the regions identified as intensive foraging zones (Fig. 2). Within individuals, geographical overlap in foraging intensity between FPT and FPD analyses was highly variable ranging from 0.26 to 0.98 with a mean overlap of 0.76 ± 0.03 (Table 1). Furthermore, mean overlap between areas of highest identified foraging activity was extremely low (0.11 ± 0.03, range: 0–0.78. Table 1). Also, individuals spent a lower proportion of time underwater in areas of high foraging activity identified by FPT than in those identified by FPD (Paired samples t-test with arcsine transformation: t_28_ = 5.357, p < 0.001; FPT: 42.5 ± 19.8%, FPD: 61.3 ± 16.9%). Inspection of tracks revealed that for some individuals, areas identified by FPT analysis as areas of high foraging intensity were actually areas where individuals were remaining relatively stationary at the surface and not diving (Fig. 3). This suggests that, for Australian fur seals, conventional FPT analysis may detect areas of increased surface activity rather than actual foraging areas. Thus, FPT analysis was deemed to be unreliable for identifying areas of intensive foraging and FPD analysis was used for further investigations.

### Geographic and behavioural variations in foraging intensity

**Fig 4:**
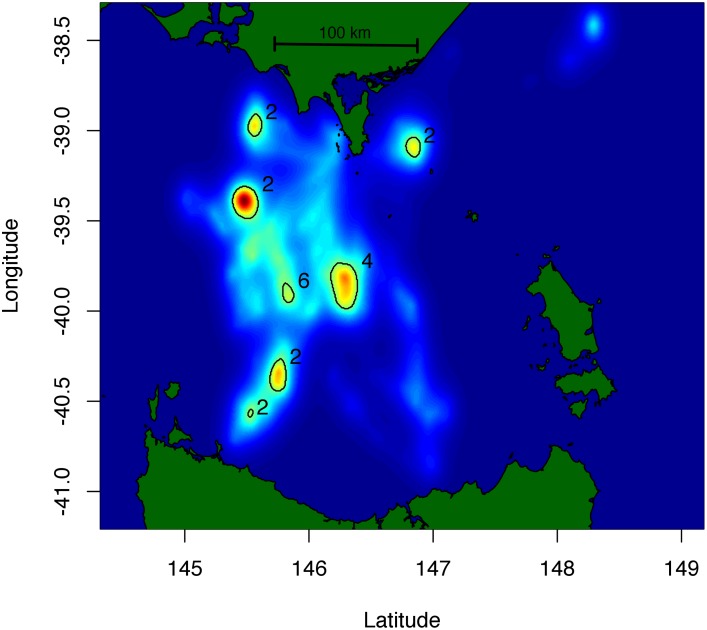
Results of a kernel home range analysis weighted by the calculated FPD foraging intensity scores for female Australian fur seals foraging from the Kanowna Island breeding colony, Bass Strait, Australia. Colour scale ramped from blue (low foraging intensity) to red (high foraging intensity). Black rings surround the core (top 10%) foraging areas and the numbers beside represent the number of individuals utilising each area.

The kernel density estimate of aggregated FPD scores revealed discreet patches of increased foraging intensity within the foraging range of Kanowna Island individuals (Fig. 4). The core areas (areas encompassing the top 10% of the density estimate) formed a mosaic of patches between 47.5 km^2^ and 168.8 km^2^ from the colony (mean: 95.1 km^2^ ± 43.5 km^2^) in areas primarily to the south-west of Kanowna Island (Fig. 4). Core foraging patch area ranged between 11.6 km^2^ and 399.2 km^2^ with a mean patch size of 158.2 km^2^ ± 135.3 km^2^ (n patches: 7).

In order to investigate fine-scale modifications in behaviour associated with areas of increased foraging intensity (as identified by the combined FPD and kernel analysis), GAMMs were developed using the scaled FPD scores as the response variable. Assessment of collinearity and variance inflation factors resulted in the removal of two (duration of bottom phase of the dive and maximum depth achieved) of the nine initial predictor variables considered for the model. Thus, the full model considered seven predictor variables, namely dive duration, total distance travelled during the bottom phase of the dive, descent/ascent rate, postdive duration, dive type, horizontal travel speed and fractal dimension. Following the backwards step-wise selection process, total distance travelled during the bottom phase was excluded, so that the most parsimonious model included only six variables (Table 2).

**Table 2:** Summary results of the Generalised Additive Mixed Effects Model used to assess effects of variation in diving and movement pattern used on foraging intensity of female Australian fur seals provisioning young at Kanowna Island, Bass Strait, Australia.

Response variables	Predictor variables	Parametric coefficients			Approximate significance of smooth terms		P
		Est	SE	t	edf	F	
Foraging intensity	Benthic dives	10.38	0.02	381.31			< 0.0001
	Pelagic dives	-0.01	0.004	-2.63			0.008
	Dive duration				6.98	28.25	< 0.0001
	Descent/ascent rate				3.84	128.53	< 0.0001
	Postdive duration				7.35	28.26	< 0.0001
	Horizontal travel speed				3.76	214.81	< 0.0001
	Fractal dimension				3.75	4912.28	< 0.0001

Est: estimated parametric coefficient. SE: estimated standard error of parametric coefficient.

**Fig 5:**
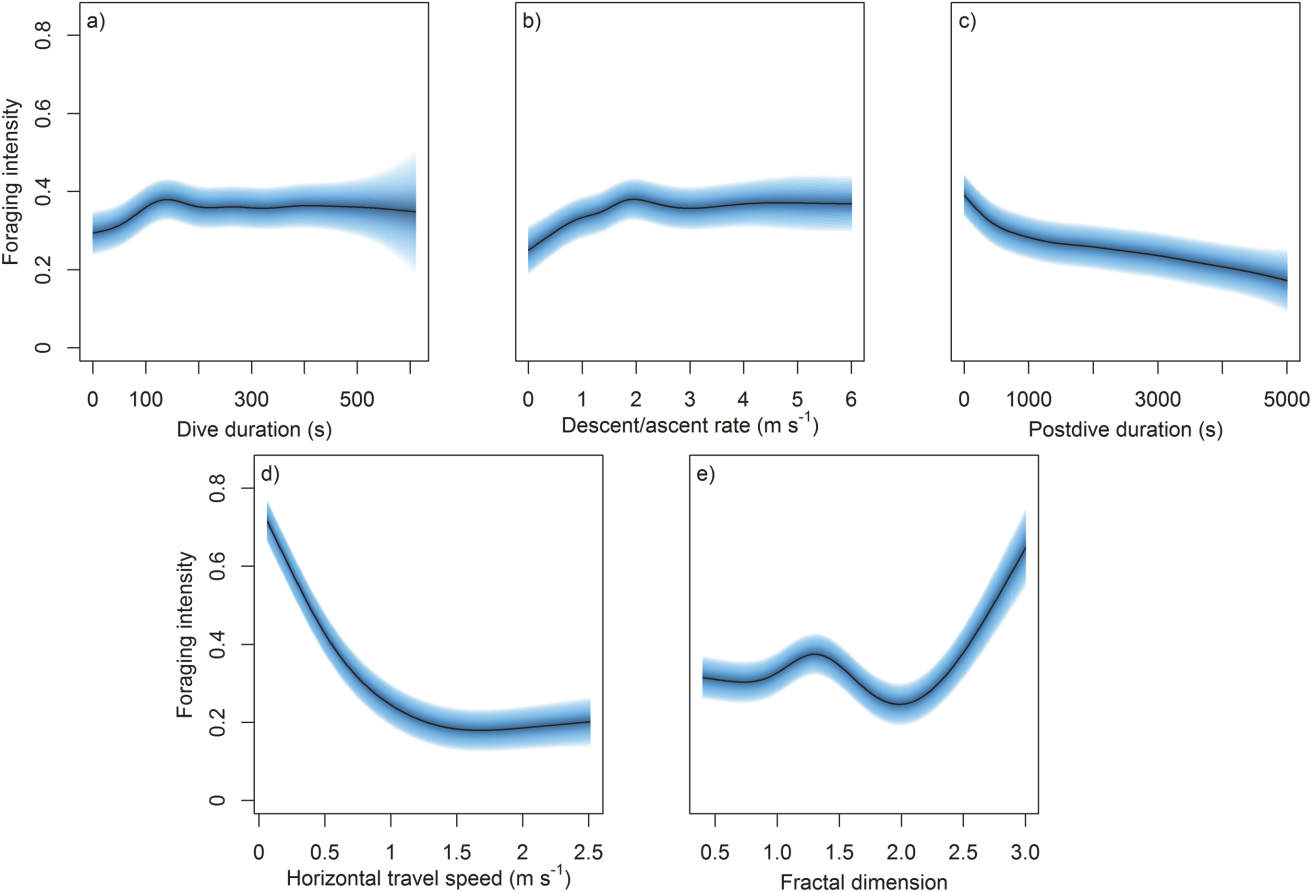
Predicted change in foraging intensity (calculated by first-passage diving analysis) in relation to variation in diving behaviour (a: dive duration, b: descent/ascent rate and c: postdive duration) and movement patterns (d: horizontal travel speed and e: fractal dimension) by female Australian fur seals foraging from the Kanowna Island breeding colony, Bass Strait, Australia. (see Table 2 for statistical results)

There was a significant but very slight (0.01% across the range of foraging intensities) increase in the proportion of benthic dives with increases in foraging intensity (Table 2). Inspection of the smooth terms showed that as foraging intensity increased, dives were most likely to last 148 s or above (Fig. 5a). Furthermore, mean descent/ascent rates peaked at 1.9 m·s^-1^ or above in areas of increased foraging intensity (Fig. 5b), while postdive duration showed the opposite trend, being reduced in areas of increased foraging intensity (Fig. 5c). Horizontal travel speeds were also lowest within areas of greatest foraging intensity with the greatest travel speeds occurring in areas with the lowest foraging intensity scores (Fig. 5d). Areas of increased foraging intensity tended to have higher fractal dimensions, i.e. 1.3, 2.3 and above (Fig. 5e). Overall this showed that, within areas of increased foraging intensity, individuals tended to minimize time at the surface and transit time to the benthos while favouring longer dives. Furthermore, by increasing their turning rate (fractal dimension) and decreasing their travelling speeds, individuals also increased their time within an area.

## Discussion

The effective identification and utilisation of profitable foraging sites by central placed foragers can significantly affect both their individual fitness and that of the offspring they are provisioning [47–50]. Numerous studies have documented the behavioural modifications of pelagic foraging species in areas of increased productivity (e.g. [51,52]) and these have been used as indices of habitat quality, aiding in the understanding of relationships between oceanographic processes, predator movements and foraging success. Comparatively little is known about how benthic foraging species alter their behaviour in relation to variations in habitat profitability [19,53–55]. In the current study, Australian fur seals were found to alter foraging effort spatially through modifications of both diving behaviour and horizontal movements.

### Identifying areas of increased foraging effort

Fauchild and Teverra’s [43] version of FPT analysis has been used extensively as a method to identify areas of increased foraging activity [15,56–58]. This method, however, relies only on location data from tracked individuals to identify areas in which animals increased their residency time. This is based on the reasonable assumption (in most cases) that it is within these areas that animals are increasing their foraging effort. However, the surface movements of air-breathing marine foragers may not fully reveal the areas of sub-surface activity [59].

Within the foraging literature a variety of other methods have been developed for the purpose of differentiating between foraging and travelling movements (e.g. fractal landscape [60], switching state-space methods, [61]and Hidden Markov Models, [62]). While these methods show promise in their ability to identify behavioural states, none have yet been developed to utilise both tracking and dive data. However, two other studies have presented modified analyses based on FPT with diving behaviour included [31,63]. Residual First Bottom Time (rFBT) relies on using/calculating per-dive residuals from a multiple regression (bottom time ~ maximum dive depth + dive duration) and using the sum of absolute values within the circle as an indicator of foraging intensity [31]. However, rFBT relies on the calculation of a foraging metric (the residuals from the multiple regression) that has been developed for southern elephant seals and not tested in other species. The second method, Spherical First Passage Time (SFPT) relies on the use of full 3D dive reconstruction (via integration of location, dive and accelerometry data) to calculate FPT values across a sphere rather than a circle [63]. This method shows promise, but currently has only been tested in simulation studies and has not been validated in a field study. As such, the application of this method to real world data remains uncertain.

The present study has shown that reliance purely on tracking data in Australian fur seals may be misleading because areas of high surface time (increased FPT score) did not reliably represent areas of increased time underwater (a measure of foraging effort). It is possible that the areas of increased surface residency (high FPT) represent areas where individuals are resting after successful feeding bouts. If true these areas should be preceded by areas of increased foraging effort (high FPD) and for some individuals this appears to be true (e.g. Fig. 3). However, for other individuals the areas of increased surface residency occur well away from areas of increased foraging effort (e.g. Fig. 2), suggesting that elevated foraging effort may not always be the cause of surface resting periods.

The accurate identification of foraging areas for a species is important when considering management decisions based on these data and misclassification of foraging areas has the potential to lead to incorrect assumptions of habitat value [64]. Arnould and Hindell [26] determined that Australian fur seals have extended diving bouts while at sea (up to 36 hours), interspersed with periods of surface activity. In the current study, such periods of surface activity, which were associated with minimal travel (e.g. Fig. 3), were incorrectly identified as important foraging areas by traditional FPT analysis, but were excluded in the FPD analysis. In southern elephant seals, the inclusion of diving activity into an FPT analysis leads to a reduction in spatial scale of the foraging behaviour being identified and differences in the location of those foraging areas [31]. While in the present study the inclusion of diving behaviour did not lead to a change in spatial scale it found that, within individuals, there was minimal overlap between the areas of high use identified by the two methods. Therefore, the results indicate that inclusion of diving behaviour into analyses is beneficial for identifying areas of increased foraging intensity in Australian fur seals, and potentially other benthic foraging pinnipeds.

Using satellite telemetry, previous studies of the foraging areas of Australian fur seal females identified several foraging “hot spots” within the Bass Strait region [24,25]. The coarse resolution of satellite telemetry, however, restricted the definition of these to broad regions of 20,173–59,678 km^2^. Arnould and Kirkwood [24] found female Australian fur seals preferentially foraged in continental shelf waters of 60–80 m depth. However because the bathymetry of central Bass Strait is fairly consistent, identifying features that individuals may be targeting within these broadly defined foraging “hot spots” is problematic without greater spatial resolution of at-sea movements.

In the present study, the combined use of high resolution (GPS) tracking and dive behaviour loggers in the FPD analysis, has enabled a greater fine-scale analysis of the distribution of foraging effort by female Australian fur seals from Kanowna Island. While the areas identified coincide with the broad “hot spots” reported in the previous satellite telemetry studies [24,25], the present study highlights a mosaic of localised important areas within the species’ foraging range. The ability to identify such specific areas may, in future studies, enable in-depth investigations of the benthic habitat features that Australian fur seals prefer to utilise as foraging grounds, either through direct survey or animal-borne methods (e.g. animal mounted cameras or oceanographic sensors).

In contrast to previous studies working with a modified FPT analysis [31,63], this analysis utilises data that are not species specific (e.g rFBT [31]) and more readily available (tracking and TDR data rather than tracking, accelerometry and TDR data). As such, this analysis shows promise in its applicability to a broad range of foraging studies. However, further validation studies should be conducted utilising value adding datasets (accelerometers or animal mounted cameras) to both assess the relationship between FPD and actual foraging success and the behaviour of this method when using data collected from different species.

### Behavioural modifications used to increase foraging effort

The FPD analysis determined where an animal was increasing its foraging effort. However, it did not determine how the animal was achieving this. Whereas, FPT analysis assumes increases in foraging activity is a result of individuals increasing their tortuosity and decreasing their speed (area-restricted search), FPD analysis developed in the present study looks for increases in underwater activity. Animals can increase their time submerged within an area by modifying diving behaviour (dive time, postdive interval etc.) as well as surface movements [30,43,65,66]. The current study determined that female Australian fur seals modified surface movements *and* diving behaviour when increasing their foraging intensity, allowing them to maximise the time within the foraging zone.

Animals in the present study increased their mean descent/ascent rates up to 1.9 m·s^-1^ with increasing FPD, while descent/ascent rates faster than this were associated with a slight reduction in FPD. Following the calculations of Feldkamp [67], the predicted minimum cost of transport velocity for the Australian fur seals in this study (average length 1.5 m) is 1.9 m·s^-1^. This suggests that, during the descent/ascent phases of dives and within areas of increased foraging intensity, individuals are increasing swim speeds up to their most efficient point and/or increasing descent and ascent angles to near 90° to minimize the time spent transiting to and from the surface.

Descent/ascent rates higher than 1.9 m·s^-1^ were most likely to be associated with elevated foraging intensity, albeit not the highest intensities, suggesting that individuals will, at times, exceed the minimum cost of transport to reach the seafloor or surface quickly. These elevated descent/ascent rates may represent individuals hurrying to access highly mobile prey spotted from above or subsequently hurrying to reach the surface after a prey capture event. Indeed animal-borne video deployments on Australian fur seals have recorded such events ([68], *Arnould unpublished data*). Furthermore, within areas of increased foraging intensity, animals minimized their time at the surface between dives. As long as the surface interval is sufficient to re-perfuse tissues with oxygen, then minimising surface intervals will allow individuals to maximise time submerged within their foraging zone [69,70]. These observations fit with optimal dive models that suggest an animal should be maximising its time within the foraging zone to optimise foraging success [69,71–73].

In zones of increased FPD, there was a slight peak in the proportion of dives with durations of approximately 148 s which is lower than the mean dive duration for the animals within this study (187 ± 63 s). With the exception of short periods of inactivity at the surface, Australian fur seals perform dives almost constantly while at sea [26,30] with a large number of dives occurring outside the identified areas of intensive foraging. Australian fur seals forage on a variety of different prey types [27,28,74]and areas where foraging intensity is the greatest may represent areas where they are targeting specific prey or specific habitats. Thus, the peak in dive durations around 148 s may represent the use of prey/habitat specific foraging tactics. In southern elephant seals, individuals have been shown to decrease bottom time and dive durations in areas of higher prey densities [75]. Dives performed in intensive foraging zones of durations greater than 148 s may represent individuals extending dive duration in order to chase mobile prey or search for more cryptic prey [21].

The energy required to chase and capture prey exceeds that required for simple locomotion [76]. Therefore, in areas where animals are chasing and capturing more prey their ability to maintain aerobic activity while underwater would be reduced, resulting in reduced dive times or increased surface times to recover from anaerobic respiration [76,77]. Furthermore, the energetic requirements of the digestion and assimilation of captured prey may also work to reduce an individual’s aerobic capacity [77]. Consequently, in areas where an animal is successfully capturing more prey, its ability to repetitively dive for longer durations may be reduced. Williams *et al* [76], for example, determined that for Weddell seals (*Leptonychotes weddellii*), the cost of a foraging dive was 44.7% greater than non-foraging dives with the costs primarily attributed to digestion and assimilation of prey. Therefore, the observed small but greater likelihood of individuals in the present study performing shorter than average dives within areas of increased foraging intensity may be the result of increased foraging success. Indeed, when assessing foraging effort at only the temporal scale, Australian fur seals have been shown to reduce dive durations across a day and over the duration a foraging trip [30], presumably as a result of the increased metabolic costs of digestion and assimilation of prey.

In the current study, Australian fur seals were actively maintaining their position within an area by increasing turning rate (fractal dimension) and decreasing travel speed. These results are consistent with individuals adopting, to a degree, an area-restricted search strategy when increasing foraging effort. Animals have been found to undergo this behaviour when foraging on patchily distributed resources to maximise foraging success and the use of this by Australian fur seals suggest some spatial heterogeneity in the prey resources available to them. Unfortunately, minimal data exist on the fine-scale benthic habitat features that exist within Bass Strait so it remains unclear what features individuals may be targeting [78].

In summary, the use of a first-passage time analysis modified to incorporate diving behaviour (FPD) proved to be more robust than a traditional FPT analysis for determining increases in foraging activity. This was in part due to the tendency of animals to spend time at the surface resting during a foraging trip. The analysis revealed a mosaic of foraging activity ‘hot-spots’ within the foraging range of animals from Kanowna Island. However, the environmental features that make up these areas are still unknown. Furthermore, Australian fur seals were found to modify both their diving behaviour and surface patterns when increasing foraging intensity suggesting that individuals are maximising benthic time within their foraging zones. Further studies assessing the composition of fine-scale benthic habitat features within the identified foraging zones would be beneficial for better understanding the ecological needs of this species.

## Supporting Information

S1 Data A compressed. RData binary file. When opened in R there is a single object named ‘data’ which is a data.frame containing the primary set of data used in this study. This consists of a table of the combined dive, tracking and IFZ statistics for each individual.
(ZIP)
